# Abrupt sand-dune accumulation at the northeastern margin of the Tibetan Plateau challenges the wet MIS3a inferred from numerous lake-highstands

**DOI:** 10.1038/srep25820

**Published:** 2016-05-13

**Authors:** Hao Long, Markus Fuchs, Linhai Yang, Hongyi Cheng

**Affiliations:** 1 State Key Laboratory of Lake Science and Environment, Nanjing Institute of Geography and Limnology, Chinese Academy of Sciences (NIGLAS), 210008 Nanjing, China; 2 Geochronology and Isotope Hydrology, Leibniz Institute for Applied Geophysics (LIAG), 30655 Hannover, Germany; 3 Department of Geography, Justus-Liebig-University Giessen, 35390 Giessen, Germany; 4 Key Laboratory of Desert and Desertification, Cold and Arid Regions Environmental and Engineering Research Institute, Chinese Academy of Sciences, 730000 Lanzhou, China; 5 College of Earth and Environmental Sciences, Lanzhou University, 730000 Lanzhou, China

## Abstract

Over the Tibetan Plateau and adjacent regions, numerous ^14^C-based lake records revealed a ubiquitous wet climatic period during 40–25 ka (late MIS 3), which is in contradiction with the global pattern of generally cold and dry climates. This paper focuses on OSL dating results of a large set of sand dunes and alluvial sediments (50 OSL ages) from the Qinwangchuan (QWC) Basin at the northeast edge of the Tibetan Plateau, with the aim to test the validity of the anomalous wet condition for the late MIS 3 interval, evidenced by numerous lake highstands. The abrupt sand dune accumulation as indication of increased aridity in the study area was OSL dated to ~40–13 ka. This dry climatic inference of the sand dune system from QWC apparently shows no wet MIS 3a event. Thus, the anomalous wet conditions revealed by high lake levels for the late MIS 3 phase may not be a universal phenomena across entire western China.

Over the past several decades, climatic and environmental changes during the last glacial period in western China, including the arid and semiarid regions of northwest China and the Tibetan Plateau, have been extensively reconstructed using records from lakes, ice cores, and loess-palaeosol units. One main controversial issue refers to an anomalous warm and humid phase between 40 and 30 ka corresponding to the late part of marine isotope stage (MIS) 3^1^. Since the 1980 s, numerous geological evidences have apparently shown palaeoclimatic conditions much warmer and wetter than today over the Tibetan Plateau and the desert areas to its north during the late MIS 3 or even early MIS 2^2^,^3^,^4^,^5^,^6^. For instance, based on an ice-core oxygen isotope record (Guliya ice core) from the western Tibetan Plateau, it has been inferred that an extremely warm climatic situation dominated at ~40–32 ka during the late MIS 3, and the peak temperature at 35 ka was estimated by Thompson *et al*.^7^ and Yao *et al*.^8^ to be ~4 °C higher than that of the present-day. The extensive wet conditions during this time period has also been unveiled according to the high lake-level stands of ~40–25 ka coupled with sedimentological proxy analysis from numerous lakes from the Tibetan Plateau^9^,^10^,^11^,^12^,^13^, as well as other neighboring desert areas^2^,^5^,^6^,^14^,^15^,^16^. Consequently, an anomalous warm and wet late interstadial within the last glacial period has been supposed for western China. Meanwhile, modeling analysis further supported this anomalous wet event^17^, and attributed its forcing mechanisms to the strengthening of the Indian summer monsoon during the period of ~40–30 ka, following the orbital precessional cycle, which brought more moisture into the Tibetan Plateau^3^, either or the strengthened westerlies which gave rise to more precipitation in northwestern China^5^,^6^.

The apparently unique regional warm and humid pattern of late MIS 3 (i.e., MIS 3a) from the western China, however, conflicts with global climatic contexts reconstructed from the SPECMAP δ^18^O record^18^, the Greenland GRIP ice core^19^ and the Antarctic Vostok ice core^20^, which all suggest the last glacial stepwise increase of global ice volumes and the gradual decrease of sea-surface temperatures toward the Last Glacial Maximum (LGM). The lake-based wet climate anomaly is also considerably inconsistent with other regional proxy records showing the late MIS 3 period was relatively drier compared with the last interglacial period and the post-glacial Holocene stage^21^,^22^,^23^,^24^,^25^,^26^. Moreover, the ^14^C dating, most commonly employed to support wet climate at the period of 40–30 ka, may suffer from the problem of saturations^27^,^28^. These controversies have led to the conclusion that the anomalously wet climate basically inferred from numerous lakes remains an enigma^29^,^30^.

Sand dune accumulation and related alluvial sediments from arid and semiarid areas have been considered as an archive of past moisture variations due to their sensitivities to alternations of regional aridity and wetness, and related atmospheric circulations^31^,^32^,^33^,^34^. Here we present a large set of luminescence ages of sand dunes and alluvial aggradations from the Qinwangchuan (QWC) Basin, situated at the northeastern edge of the Tibetan Plateau, currently influenced by the Asian monsoon systems, resulting in a warm and moist summer monsoon and a cold and dry winter monsoon (Fig. 1a). Our chronostratigraphic and palaeoclimatic interpretations of the investigated sediment archives provide an opportunity to examine the hypothesis of an anomalous wet period during the late MIS 3, so far dominantly derived from lake records.

## Results

In combination of all investigated sections, the chronostratigraphy of a large set of sedimentary sequences from the QWC Basin is shown in Fig. 2. With the exception of several dates, presented age estimates from each profile are stratigraphically sensible with minor inversions in ages lying within errors. Age-depth profiles are not the same between the eight dune sections shown here (XC1, XC2, XC3, XC4, XC5, XC6, ZC2, and ZC3). This indicates that individual dune profiles are not able to in fact record aeolian accumulation phases with a comprehensive temporal and spatial significance for the study area, which is due to common unconformities and discontinuities in aeolian sediments^31^,^32^. We thus compile a composite sand dune accumulation record from these multiple study sites by putting all OSL dates from the dunes together (Fig. 3a). The youngest age (14.8 ± 1.4 ka) is from profile ZC3, suggesting that the aeolian sand accumulation in the study area has been interrupted after that time. The upper limit of the sand dune formation is documented by sample XC1–13 and extends back to 37.3 ± 3.1 ka, according to the new dates from the base of profile XC1. Overall, all obtained dates indicate a solely abrupt accumulation of aeolian sand dune in the southern margin of the QWC Basin at approximately ~40–13 ka (grey bar in Fig. 3).

**Figure 1:**
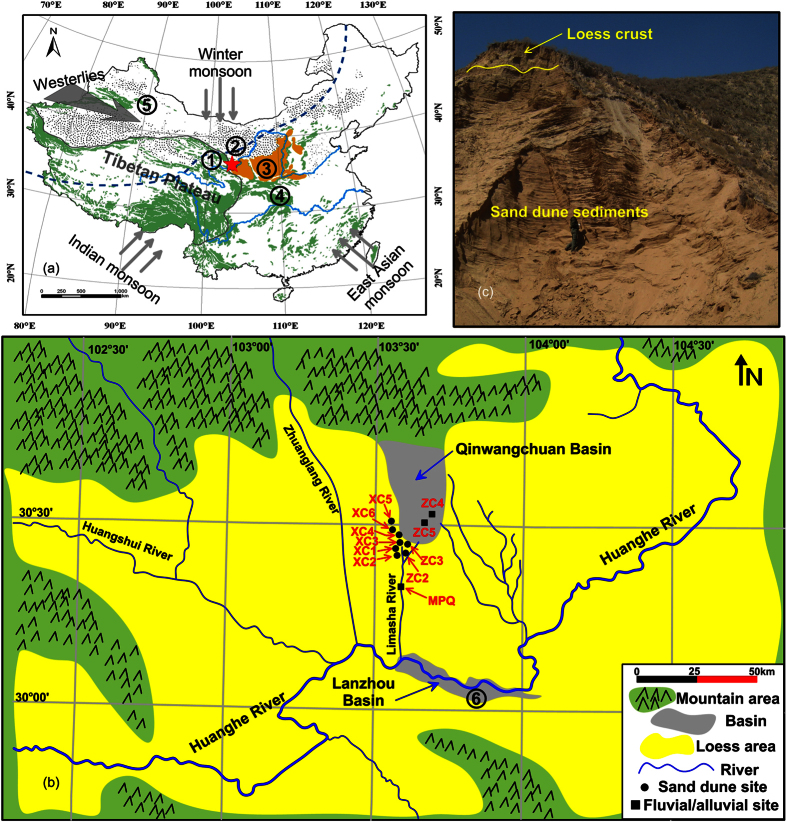
(**a**) Map shows the northern limit (blue dashed line) of the modern summer monsoon (according to Long *et al*.^25^), and the dominant circulation systems of the Westerlies, the Indian monsoon, and the East Asian summer and winter monsoon; the red star indicates the location of the QWC Basin, and the numbers show the location of palaeoclimatic records cited in this paper for comparison (① Qinghai Lake^23^,^48^, ② Tengger Desert^14^,^15^,^26^, ③ Xifeng profile^45^, ④ Sanbao Cave^44^, ⑤ Balikun Lake^56^,^57^, ⑥ Lanzhou Basin^24^. This map was generated using ArcGIS 10.2.2 (http://www.esri.com/software/arcgis). (**b**) Geomorphological map of the study area (according to Yuan *et al*.^58^) and sampling localities. This map was drawn with CorelDraw X4 (http://www.coreldraw.com). (**c**) An outcrop example (XC1) showing typical sand dune sediments and overlying loess.

**Figure 2:**
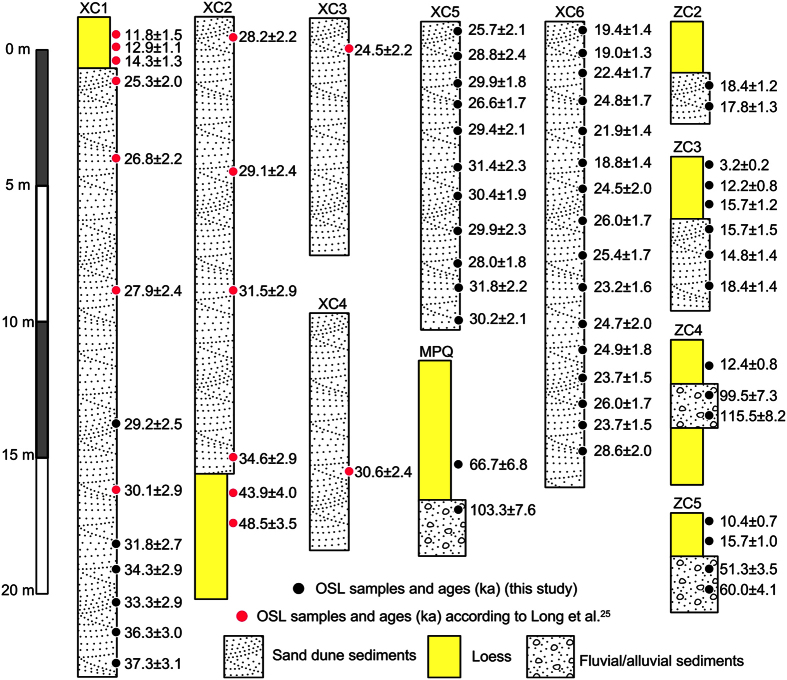
Stratigraphy and chronology of all investigated profiles.

**Figure 3:**
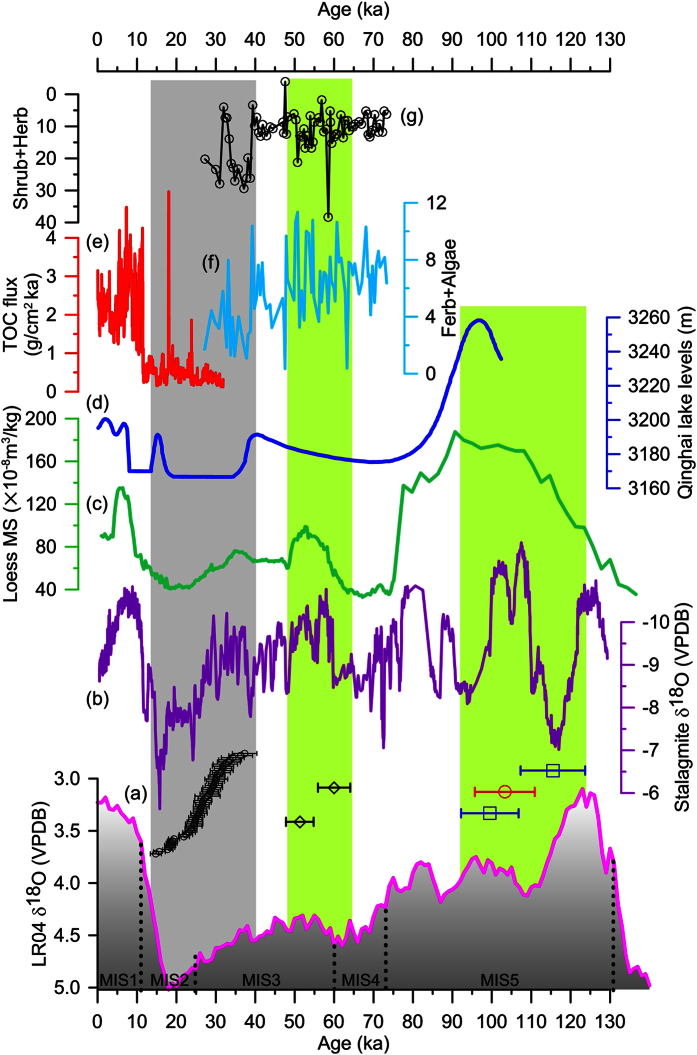
Comparison of different records (grey bar shows the interval of abrupt sand dune accumulation in QWC Basin and regional drought event, and green bars show the periods of increased fluvial activity and regional moisture) with marine oxygen isotope stages according to LR04 stack^1^: (**a**) OSL age clusters for sand dune sediments (black circle) and alluvial sediments from ZC5 (black diamond), ZC4 (blue square), and MPQ (red circle); (**b**) Chinese stalagmite oxygen isotope composite records indicating monsoon precipitation variations (negative represents strengthened monsoon and increased precipitation, and vice versa)^44^; (**c**) Xifeng MS record (high MS indicates increased regional moisture and strong summer monsoon, and vice versa)^45^; (**d**) Lake levels reconstruction based on OSL dating of lake shorelines from Qinghai Lake^23^; (**e**) Total organic carbon (TOC) flux records monsoonal precipitation in Qinghai Lake during the past 32 ka (high TOC flux reflects strengthened monsoonal precipitation, and vice versa)^48^; (**f,g**) are pollen records from Lanzhou Basin (low concentrations of Ferb + Algae and high concentrations of Shrub + Herb represent dry conditions, and vice versa)^24^.

The central part of the QWC Basin is dominated by alluvial sediments. The sandy gravel unit in ZC5 yields two ages of 60.0 ± 4.1 ka and 51.3 ± 3.5 ka, while the overlying loess provides ages of 15.7 ± 1.0 ka and 10.4 ± 0.7 ka (Fig. 2 and Text S1). A little further to the south, a sandy pebble alluvium intercalated with loess (ZC4, Fig. 1b) gave much older OSL ages of 115.5 ± 8.2 ka and 99.5 ± 7.3 ka, and the upper loess was dated to 12.4 ± 4.1 ka (Fig. 2). In the 20 km further to the middle reaches of the Limasha River (Fig. 1b), an outcrop (MPQ) of ancient river bed sediments is dated to 103.3 ± 7.6 ka (Fig. 2 and Text S1), which is consistent with the age of alluvial sediments in ZC4. Based on the chronostratigraphy of these three sections, fluvial sedimentation dates to MIS 5 (Fig. 3a), both inside and outside of the QWC Basin (ZC4 and MPQ, Fig. 1b), probably suggesting a regional increase of fluvial activity. Another fluvial section ZC5, stretching about 1000 m along an ancient river bed, probably indicates another episodic fluvial sedimentation event during the early MIS 3 (Fig. 3a). Loess from the upper part of ZC4 and ZC5 shows contemporaneous dust accumulation since the late glacial as loess crusts overlying sand dune sediments in XC1 and ZC3 (Fig. 2) located at the southern edge of QWC Basin (Fig. 1b).

## Discussion

### Palaeoclimatic interpretations of sand dune accumulation and alluvial activity in the QWC Basin

The formation of sand dunes is often interpreted as regional aridity in arid and semiarid environments^31^,^35^,^36^,^37^. Nevertheless, recent investigations of aeolian archives from the northeastern Tibetan Plateau suggest that the connection between climate and sand dune system response might not be straightforward^38^, and even some similar sand dune archives may record different climatic conditions^39^. Besides the dry climatic conditions, therefore, other primary factors controlling sand dune formation, e.g., sand availability in the source area, wind conditions, and local situations of the deposition areas^34^,^40^,^41^, should also be considered to further understand the drivers and palaeoclimatic implications of sand dune occurrences. Firstly, the modern observation showed that the present-day strong winds are capable of sweeping from north to south across the QWC basin on many days during winter time^42^, which easily results in deflation. Furthermore, the regional monsoon regime, northerly and northwesterly winds dominant in winter time, has been thought to be significantly strengthened during the last glacial period according to geological records from the Chinese loess plateau^43^. Thus, the requisite in wind conditions can be met for forming dunes downwind of the basin. Secondly, the surrounded loess hills provide an ideal topographic barrier to sand transport. Third, sandy sediment supply is a basic requirement for dune development^34^, and in the central QWC Basin, extensive sandy alluvial deposits from ancient streams are obvious potential sources for providing sands. Our OSL dating results of subsurface sediments from ZC4 and ZC5 suggested fluvial appearance at the early MIS 3 as well as in MIS 5 period, which may supply massive amounts of sands for subsequent dune formation during ~40–13 ka. In addition to the possible sand source, nevertheless, conditions that can set the dry, loose, bare sand to motion when wind blows should be equally critical for sand dune accumulation downwind^25^. We thus believe that an arid climate should be dominant, triggering a dry and unvegetated fluvial plain in QWC. For these reasons, the abrupt dune development newly dated to ~40–13 ka can presumably be interpreted as increased aridity during that time in the QWC Basin. In contrast, the two fluvial sedimentary intervals (green bars in Fig. 3) may be correlated with a significant increase in monsoonal precipitation at the early MIS 3 and the MIS 5 as recorded in oxygen isotope records of Chinese stalagmites (Sanbao Cave)^44^ (Figs 1a and 3b) as well as in the loess magnetic susceptibility (MS) proxy record from Xifeng profile on the Chinese loess plateau^45^ (Figs 1a and 3c), and thus the fluvial sedimentation may represent dominant wet condition.

### Challenging the anomalous wet event inferred from lake records

The dry climatic condition during the period of ~40–13 ka, revealed in the sand dune record from QWC, is apparently challenging the major hypothesis of wet MIS 3a mostly based on numerous lake highstands over the Tibetan Plateau and its surrounding areas. Nevertheless, the validity of a none wet MIS 3a has seemingly been supported by other proxy evidences. A pollen-based vegetation record of the last glacial period from Lanzhou Basin, ~40 km south of QWC Basin, indicated a climatic deterioration and drought since 40 ka^24^ (Figs 1a and 3f,g). Moreover, the finding of a dry event inferred from the sand dune formation in the current study can be verified by magnetic susceptibility variations of a series of loess-palaeosol sequences across the Chinese loess plateau, e.g., Xifeng profile^45^ (Figs 1a and 3c), which did not show any anomalous strengthening of the monsoonal precipitation and, in turn, regional moisture during the MIS 3 period^43^,^46^,^47^. Recently, a lake core was retrieved from the deposition-center of Qinghai Lake (Fig. 1a), the largest lake on the Tibetan Plateau, and its lithological data showed that this core is composed of loess-like and fine sand sediments, indicating very shallow lake environment and the lake even dried out with considerable aeolian input during ~32–20 ka^48^. Meanwhile, proxy data of this core further confirmed a weak Asian monsoon influence and dry/cold conditions dominating at that time^48^ (Fig. 3e). These results are generally in agreement with the climatic inference in QWC Basin, but in contrast to the earlier report that a highest lake terrace formed at 40–30 ka around Qinghai Lake^49^,^50^, which has been attributed to increased regional precipitation and river runoff^51^ according to the energy-balance model of Kutzbach^52^.

It is noted that the anomalous wet event for late MIS 3 is basically derived from ^14^C dated fragmentary shorelines and terraces around lakes across the Tibetan Plateau and adjacent areas, which indicate lake levels above the present-day conditions^12^,^29^. During the last decade, there have been more and more interests in applying dating techniques other than the radiocarbon method, to determine the ages of high lake-level events. These dating results did not support the occurrence of high lake levels for ~40–30 ka. For instance, a set of OSL dates from lake shorelines over the northeastern margin of the Tibetan Plateau found that the highstands formerly dated back to MIS 3 interval by ^14^C method are placed to the period beyond ~70 ka^23^,^26^,^30^. Madsen *et al*. reconstructed lake level histories of Qinghai Lake by OSL dating of lake shorelines and terraces, suggesting that the maximum highstands date to MIS5 but MIS3 highstands are much lower^23^ (Fig. 3d). Similarly, the mega-lake period in the Tengger Desert (Fig. 1c), originally postulated as MIS 3 interval by numerous ^14^C dates, is dated to MIS 5 phase with OSL ages of ~100–70 ka^26^. More recently, Long and Shen summarized the application of different kind of dating techniques in age control of high lake levels, and argued that the timing of the late Pleistocene lake highstand is likely to be significantly underestimated by ^14^C dating techinique^27^. Furthermore, the proposed MIS 3a mega-lake event, as indication of extensive wet environment, has further been challenged in chronological aspects by a comprehensive OSL and ^14^C dating comparison of a late Quaternary lacustrine sequence from Xingkai Lake, NE Asia^28^. This study explored the possible causes for ^14^C age underestimation beyond 30 ka, and then highlighted that a combination of proximity to the radiocarbon limit, causing the indistinguishable ^14^C activity of sample from the background, and/or contamination with very small amounts of modern carbon may explain why many lake sediments representing highstands yield similar ages at the range of ~40–30 ka. Therefore, the previous inference of anomalous wet events according to such old radiocarbon dates beyond ~30 ka may be erroneous.

The mechanism responsible for the postulated late MIS 3 moist interval is open to debate^24^,^29^. A traditional point of view ascribed this event to the strengthening of the Asian monsoon driven by the precession cycle^3^, based on that the precessional cycle during ~40–30 ka shows strong summer insolation at 30° N^53^, which results in: (1) the increased heat source over the Tibetan Plateau not only facilitating the northward shift of the Indian monsoon from the southern slope of the plateau, but also strengthening the extension of the East Asian monsoon northwestwards the arid region of northwest China; (2) this enhances temperature of equatorial oceans and intensified evaporation from their surface, causing more amounts of moisture available to be carried to the Tibetan Plateau and adjacent regions. Both aspects could give rise to more moisture being transported to most of the areas in western China. However, most monsoon records have not shown any strengthening between 40 and 30 ka. Paleoceanographic data reconstruction from a marine core in the Bay of Bengal suggested higher salinity during 40–15 ka and hence decreased monsoon precipitation^54^. A recent speleothem δ^18^O record from Xiaobailong cave in southwest China characterizes changes in summer precipitations in the Indian monsoon dominant areas^55^, which resembles the salinity reconstruction of Kudrass *et al*.^54^ and shows no increased precipitation at the late MIS 3. On the other hand, Yang *et al*. argued against the monsoonal forcing, and highlighted that a moist climate during MIS 3, especially in the arid northwest China and north of Tibetan Plateau, could be better explained by the strengthened westerlies^5^, because the significant expansion of the northern ice sheets since ~40 ka increased the polar-to-equator temperature and pressure gradients and then strengthened the westerlies, supplying moisture to many places in northwest China during 40–30 ka. Lake sediment records from Balikun Lake in Xinjiang, where climate conditions are dominantly controlled by the westerlies, may be used to test the impact of westerlies on moisture variations and lake levels. In conflict with high lake levels, however, an earlier reconstruction of the carbonate δ^18^O record from this lake implied a gradual aridification trend from MIS 3 to LGM^56^, and recently published high-resolution lacustrine pollen and grain-size records from Balikun Lake showed similar results^57^.

Similar to our inference from QWC Basin, therefore, variable proxy records do not show the humid MIS 3a interval mostly inferred from dated lake sediments, which indicate lake levels above the present-day conditions. This anomalous event may not be universal across entire western China, or its ^14^C-based chronology is questionable. Better age control for lake highstands, as primary indication of wet climate, is still required.

## Conclusions

This study presented a large set of OSL ages of last glacial sand dune and alluvial sediments from the QWC Basin at the northeast edge of Tibetan Plateau. An abrupt sand dune accumulation was dated to ~40–13 ka, interpreted as a dry climate in the study area, and two fluvial activities are identified in late MIS 5 and early MIS 3, which may supply sand source for the occurrence of sand dune accumulation in the southern QWC Basin. The dry climatic condition during the ~40–13 ka revealed in the sand dune record from QWC, is apparently challenging the major hypothesis of wet MIS 3a mostly based on numerous lake highstands over the Tibetan Plateau and northwest China. The validity of none wet conditions during the 40–30 ka has also been supported by other proxy evidences. Thus, we conclude that the anomalous wet conditions and high lake levels for late MIS 3 interval may not be ubiquitous across the entire western China.

## Materials and Methods

The QWC Basin, located at the northeastern margin of the Tibetan Plateau (Fig. 1a), has an average elevation of ~2000 m a.s.l., and extends north-south for ~42 km and east-west for ~20 km. Geophysical surveys in the basin have shown the faulted nature of the graben basin along two lateral faults. The subsurface stratigraphy in logged by wells and boreholes show that the basin fillings consists mainly of sands and pebbly sands of fluvial or alluvial origin, concealed beneath a thin loess layer^58^. Except for the north characterized by mountains of volcanic origin, the basin is flanked by loess hills on the east, west and south, and drained by the intermittent river Limasha, which flows south into the river Huang He (Yellow River) (Fig. 1b).

Chen *et al*. reported first for sand dune formation in the southern margin of the QWC Basin^49^. As far as recently, the dune accumulation chronologies of ~35–25 ka have been preliminary built up by Long *et al*. using optically stimulated luminescence (OSL) dating of several outcrops^25^ (XC1, XC2, XC3, and XC4, Fig. 1b for their locations) where typical dune sediments are exposured, e.g., XC1 (Fig. 1c). Considering the nature of discrete sedimentation of dunes, nevertheless, our ability to identify the complete timing of sand dune accumulations may be hindered by having either a small number of ages derived from each of multiple profiles (i.e., low-sampling resolution) or relatively limited number of sampling sites^59^. For this reason, we revisited one formerly investigated outcrop XC1 where we had previously obtained seven OSL dates^25^, and six new samples were collected from the lower part of dune sand layer, potentially providing the onset age of sand dune accumulation; four profiles (XC5, XC6, ZC2, and ZC3, Fig. 1b for their locations and Text S1 for site XC6) including typical sand dune sediments have newly been investigated, and for each site sampling at around 1 m vertical intervals was conducted to produce a high-sampling resolution record of dune accumulation. In addition, three fluvial/alluvial, and related, sequences (ZC4, ZC5, and MPQ, Fig. 1b for their locations and Test S1 for sites ZC5 and MPQ) from the vicinity of stream channels were used to reconstruct the possible fluvial processes in QWC Basin.

A total of 50 samples were taken for OSL dating in this study. Sample collection, pretreatment and measurement for OSL dating follow Long *et al*.^25^. OSL measurements were performed using an automated Risø TL/OSL-20 reader. Stimulation was carried out by a blue LED (470 nm) stimulation source for 40 s at 130 °C. Irradiation was carried out using a ^90^Sr/^90^Y beta source built into the reader. The OSL signal was detected by a 9235QA photomultiplier tube through a 7.5 mm thick U-340 filter. OSL signals from the first 0.64 s stimulation were integrated for growth curve construction after background subtraction using the last 50 channels in the shine-down curve. The D_e_ was determined using the single aliquot regenerative dose (SAR) protocol^60^. Radionuclide activity concentrations were determined from measurements of U, Th and K concentrations using neutron activation analysis (NAA) of dried and ground bulk samples.

Additional information on stratigraphical description of the investigated profiles and all OSL chronologies are presented in Text S1 in the supporting information.

## Additional Information

**How to cite this article**: Long, H. *et al*. Abrupt sand-dune accumulation at the northeastern margin of the Tibetan Plateau challenges the wet MIS3a inferred from numerous lake-highstands. *Sci. Rep.* **6**, 25820; doi: 10.1038/srep25820 (2016).

## Supplementary Material

Supplementary Information
