# Supplementary material for: Abrupt sand-dune accumulation at the northeastern margin of the Tibetan Plateau challenges the wet MIS3a inferred from numerous lake-highstands
Source: Sci Rep. 2016 May 13;6:25820. doi: 10.1038/srep25820 (PMC4865950; doi:10.1038/srep25820)
Supplement: Supplementary Information [file srep25820-s1.docx]

Supplementary Information

**Abrupt sand-dune accumulation at the northeastern margin of the Tibetan Plateau challenges the wet MIS3a inferred from numerous lake-highstands**

Hao Long^1, 2^, Markus Fuchs^3^, Linhai Yang^4^ & Hongyi Cheng^5^

^1^State Key Laboratory of Lake Science and Environment, Nanjing Institute of Geography and Limnology, Chinese Academy of Sciences (NIGLAS), 210008 Nanjing, China. ^2^Geochronology and Isotope Hydrology, Leibniz Institute for Applied Geophysics (LIAG), 30655 Hannover, Germany. ^3^Department of Geography, Justus-Liebig-University Giessen, 35390 Giessen, Germany. ^4^Key Laboratory of Desert and Desertification, Cold and Arid Regions Environmental and Engineering Research Institute, Chinese Academy of Sciences, 730000 Lanzhou, China. ^5^ College of Earth and Environmental Sciences, Lanzhou University, 730000 Lanzhou, China. Correspondence and requests for materials should be addressed to H.L. ([longhao@niglas.ac.cn](mailto:longhao@niglas.ac.cn); [lzugeolh@gmail.com](mailto:lzugeolh@gmail.com))

1. Description of representative sections
2. XC6 site


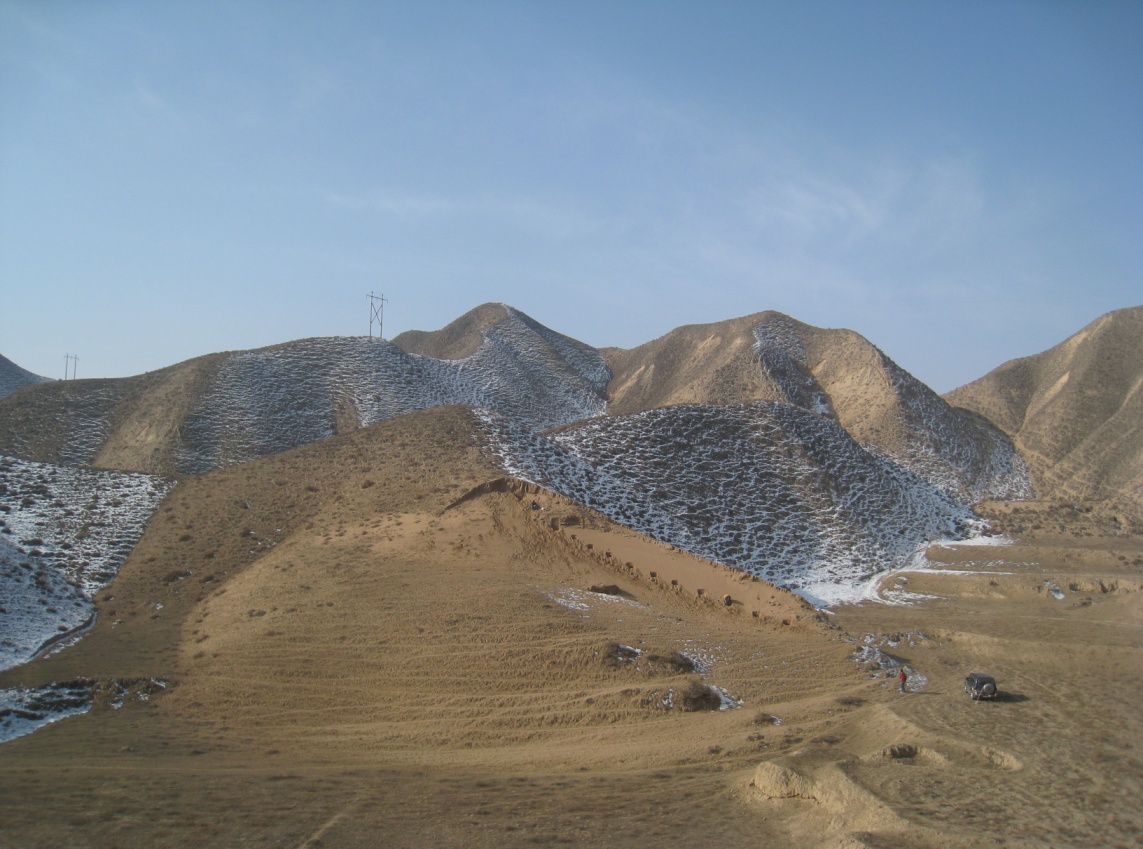


Loess

Sand dune


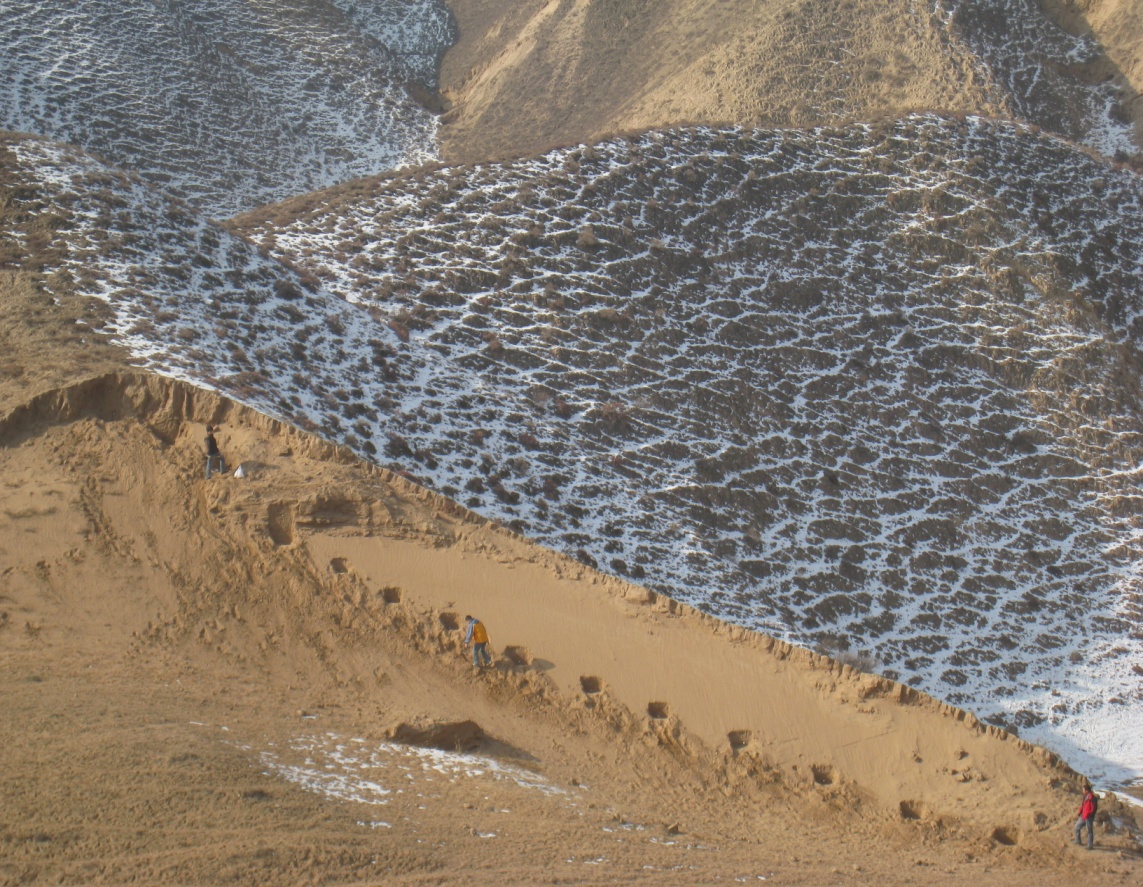


Sampling steps


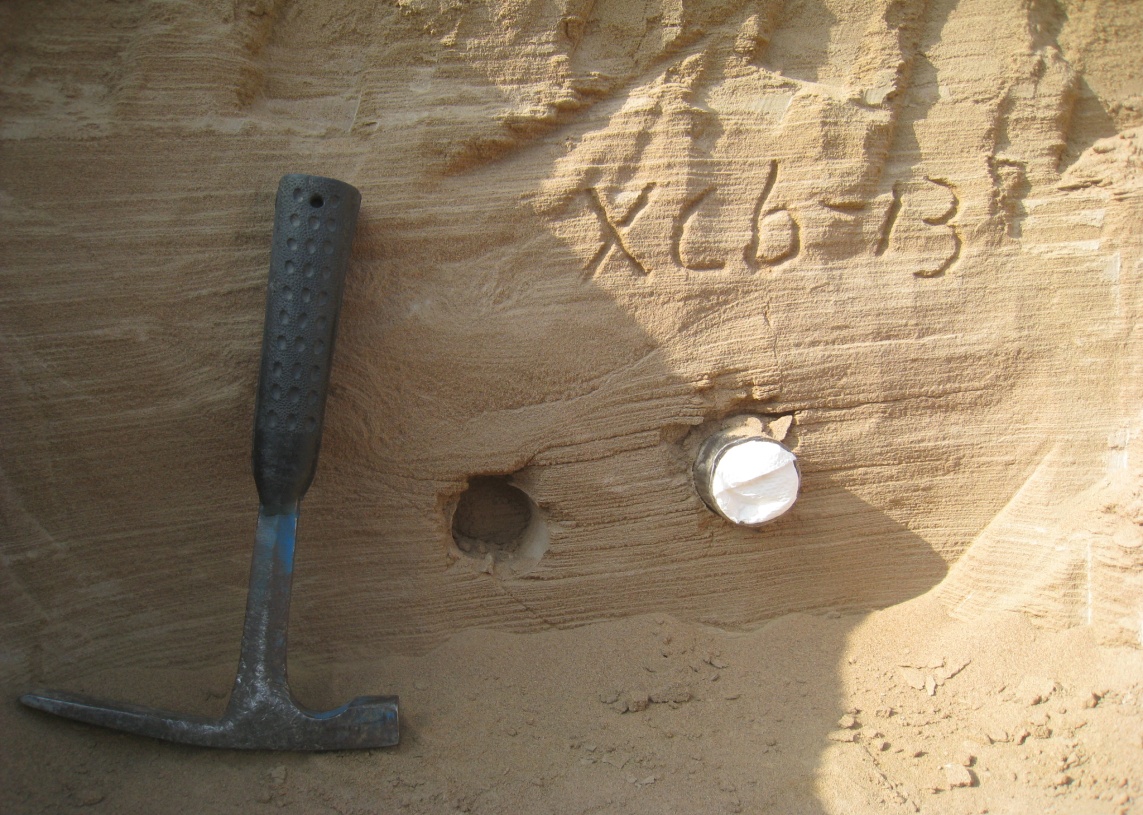


1. ZC5 site

1. MPQ site

OSL dating results for 50 samples

| Sample ID | Depth (m) | K (%) | Th (ppm) | U (ppm) | Water content (%) | Total dose rate (Gy/ka) | D_e_ (Gy) | Age (ka) |
| --- | --- | --- | --- | --- | --- | --- | --- | --- |
| XC1-8 | 14.4 | 1.00±0.12 | 4.42±0.18 | 1.29±0.05 | 5±2.5 | 1.9±0.1 | 55.4±3.4 | 29.2±2.5 |
| XC1-9 | 19.5 | 1.39±0.13 | 4.95±0.19 | 1.4±0.05 | 5±2.5 | 2.1±0.1 | 68.0±4.3 | 31.8±2.7 |
| XC1-10 | 20.2 | 1.65±0.18 | 6.34±0.25 | 1.53±0.05 | 5±2.5 | 2.4±0.1 | 83.7±5.1 | 34.3±2.9 |
| XC1-11 | 21.0 | 2.11±0.15 | 7.95±0.20 | 1.4±0.08 | 5±2.5 | 2.6±0.2 | 85.7±5.4 | 33.3±2.9 |
| XC1-12 | 22.0 | 1.44±0.14 | 5.55±0.16 | 1.37±0.07 | 5±2.5 | 2.2±0.1 | 78.7±4.3 | 36.3±3.0 |
| XC1-13 | 23.0 | 1.08±0.1 | 5.06±0.14 | 1.21±0.06 | 5±2.5 | 1.9±0.1 | 69.8±3.9 | 37.3±3.1 |
| XC5-1 | 1.0 | 1.23±0.07 | 5.91±0.16 | 1.48±0.12 | 5±2.5 | 2.3±0.1 | 57.5±3.5 | 25.7±2.1 |
| XC5-2 | 2.0 | 1.24±0.07 | 5.77±0.16 | 1.48±0.11 | 5±2.5 | 2.2±0.1 | 63.7±4.0 | 28.8±2.4 |
| XC5-3 | 3.0 | 1.22±0.06 | 5.69±0.15 | 1.50±0.12 | 5±2.5 | 2.2±0.1 | 63.9±1.2 | 29.9±1.8 |
| XC5-4 | 4.0 | 1.34±0.07 | 5.95±0.16 | 1.51±0.12 | 5±2.5 | 2.3±0.1 | 58.2±1.2 | 26.6±1.7 |
| XC5-5 | 5.0 | 1.43±0.08 | 6.79±0.18 | 1.57±0.12 | 5±2.5 | 2.5±0.1 | 69.1±2.4 | 29.4±2.1 |
| XC5-6 | 6.0 | 1.43±0.08 | 6.33±0.17 | 1.59±0.12 | 5±2.5 | 2.4±0.1 | 73.6±3.2 | 31.4±2.3 |
| XC5-7 | 7.0 | 1.40±0.07 | 5.54±0.09 | 1.65±0.10 | 5±2.5 | 2.3±0.1 | 68.4±0.7 | 30.4±1.9 |
| XC5-8 | 8.0 | 1.49±0.08 | 6.01±0.16 | 1.56±0.13 | 5±2.5 | 2.2±0.1 | 69.3±3.0 | 29.9±2.3 |
| XC5-9 | 9.0 | 1.52±0.08 | 7.62±0.19 | 1.72±0.15 | 5±2.5 | 2.6±0.2 | 72.9±1.5 | 27.2±1.7 |
| XC5-10 | 10 | 1.30±0.13 | 5.58±0.15 | 1.17±0.06 | 5±2.5 | 2.0±0.1 | 63.1±2.1 | 31.8±2.2 |
| XC5-11 | 11 | 1.22±0.08 | 4.97±0.12 | 1.34±0.07 | 5±2.5 | 2.1±0.1 | 62.3±1.9 | 30.2±2.1 |
| XC6-1 | 1.0 | 1.37±0.08 | 6.64±0.17 | 2.02±0.16 | 5±2.5 | 2.6±0.1 | 49.7±2.3 | 19.4±1.4 |
| XC6-2 | 2.0 | 1.50±0.08 | 6.86±0.18 | 1.70±0.14 | 5±2.5 | 2.6±0.1 | 49.4±1.8 | 19.0±1.3 |
| XC6-3 | 3.0 | 1.47±0.08 | 5.73±0.16 | 1.36±0.14 | 5±2.5 | 2.4±0.1 | 53.1±2.3 | 22.4±1.7 |
| XC6-4 | 4.0 | 1.43±0.08 | 6.89±0.18 | 1.38±0.13 | 5±2.5 | 2.4±0.1 | 59.7±2.0 | 24.8±1.7 |
| XC6-5 | 5.0 | 1.11±0.07 | 5.52±0.16 | 1.71±0.14 | 5±2.5 | 2.1±0.1 | 46.6±1.3 | 21.9±1.4 |
| XC6-6 | 6.0 | 1.62±0.09 | 7.06±0.18 | 1.81±0.16 | 5±2.5 | 2.7±0.2 | 50.2±2.1 | 18.8±1.4 |
| XC6-7 | 7.0 | 1.19±0.06 | 5.20±0.15 | 1.51±0.14 | 5±2.5 | 2.0±0.1 | 49.6±2.7 | 24.5±2.0 |
| XC6-8 | 8.0 | 1.22±0.07 | 5.25±0.15 | 1.18±0.13 | 5±2.5 | 2.0±0.1 | 50.8±1.2 | 26.0±1.7 |
| XC6-9 | 9.0 | 1.27±0.07 | 6.02±0.17 | 1.28±0.13 | 5±2.5 | 2.1±0.1 | 53.1±1.1 | 25.4±1.7 |
| XC6-10 | 10.0 | 1.19±0.06 | 5.64±0.16 | 1.38±0.13 | 5±2.5 | 2.0±0.1 | 45.6±1.4 | 23.2±1.6 |
| XC6-11 | 11.0 | 1.26±0.07 | 5.85±0.16 | 1.36±0.13 | 5±2.5 | 2.1±0.1 | 50.6±2.4 | 24.7±2.0 |
| XC6-12 | 12.0 | 1.16±0.07 | 5.45±0.15 | 1.77±0.12 | 5±2.5 | 2.1±0.1 | 51.6±1.7 | 24.9±1.8 |
| XC6-13 | 13.0 | 1.28±0.07 | 5.60±0.15 | 1.61±0.13 | 5±2.5 | 2.1±0.1 | 50.7±1.0 | 23.7±1.5 |
| XC6-14 | 14.0 | 1.35±0.08 | 6.25±0.16 | 1.72±0.14 | 5±2.5 | 2.3±0.1 | 59.5±0.9 | 26.0±1.7 |
| XC6-15 | 15.0 | 1.45±0.08 | 6.61±0.17 | 1.64±0.15 | 5±2.5 | 2.4±0.1 | 56.5±0.9 | 23.7±1.5 |
| XC6-16 | 16.0 | 1.33±0.08 | 5.81±0.16 | 1.34±0.14 | 5±2.5 | 2.1±0.1 | 60.7±1.6 | 28.6±2.0 |
| ZC2-1 | 2.5 | 0.84±0.05 | 3.43±0.10 | 0.92±0.09 | 5±2.5 | 1.5±0.1 | 27.8±0.9 | 18.4±1.2 |
| ZC2-2 | 3.5 | 1.29±0.07 | 5.33±0.14 | 1.21±0.10 | 5±2.5 | 2.1±0.1 | 38.0±1.5 | 17.8±1.3 |
| ZC3-1 | 1.0 | 1.62±0.08 | 9.72±0.23 | 2.42±0.16 | 7.5±2.5 | 3.1±0.2 | 10.1±0.5 | 3.2±0.2 |
| ZC3-2 | 2.0 | 1.52±0.08 | 8.96±0.22 | 2.55±0.16 | 7.5±2.5 | 3.0±0.2 | 36.3±1.1 | 12.2±0.8 |
| ZC3-3 | 3.0 | 1.22±0.08 | 5.81±0.16 | 1.46±0.12 | 7.5±2.5 | 2.1±0.1 | 33.4±1.5 | 15.7±1.2 |
| ZC3-4 | 4.0 | 0.86±0.06 | 4.86±0.14 | 1.19±0.11 | 5±2.5 | 1.7±0.1 | 26.4±2.0 | 15.7±1.5 |
| ZC3-5 | 5.0 | 1.03±0.07 | 4.53±0.12 | 1.16±0.11 | 5±2.5 | 1.8±0.1 | 26.4±2.0 | 14.8±1.4 |
| ZC3-6 | 6.0 | 1.04±0.06 | 3.63±0.11 | 0.66±0.09 | 5±2.5 | 1.6±0.1 | 28.9±1.0 | 18.4±1.4 |
| ZC4-1 | 1.0 | 1.36±0.08 | 7.93±0.20 | 1.76±0.13 | 7.5±2.5 | 2.6±0.1 | 31.8±1.1 | 12.4±0.8 |
| ZC4-2 | 2.0 | 1.09±0.08 | 6.46±0.17 | 1.90±0.13 | 5±2.5 | 2.3±0.1 | 226.5±10.0 | 99.5±7.3 |
| ZC4-3 | 3.0 | 1.35±0.06 | 6.25±0.14 | 1.86±0.14 | 5±2.5 | 2.3±0.1 | 261.6±10.7 | 115.5±8.2 |
| ZC5-1 | 0.5 | 1.91±0.11 | 10.46±0.25 | 2.90±0.19 | 7.5±2.5 | 3.6±0.2 | 37.1±1.1 | 10.4±0.7 |
| ZC5-2 | 1.0 | 1.67±0.09 | 10.13±0.24 | 2.66±0.16 | 7.5±2.5 | 3.2±0.2 | 50.9±1.7 | 15.7±1.0 |
| ZC5-3 | 1.5 | 1.51±0.10 | 9.01±0.23 | 2.20±0.18 | 5±2.5 | 3.0±0.2 | 151.6±5.6 | 51.3±3.5 |
| ZC5-4 | 2.0 | 1.57±0.09 | 7.51±0.11 | 2.21±0.12 | 5±2.5 | 2.9±0.2 | 172.6±6.1 | 60.0±4.1 |
| MPQ1 | 4.0 | 1.10±0.06 | 5.14±0.14 | 0.94±0.10 | 5±2.5 | 1.8±0.1 | 190.1±7.9 | 103.3±7.6 |
| MPQ2 | 5.5 | 1.80±0.14 | 10.91±0.26 | 3.27±0.18 | 7.5±2.5 | 3.5±0.2 | 231.8±18.4 | 66.7±6.8 |
